# Incorporating verbal and nonverbal aspects to enhance a model of patient communication in cancer care: A grounded theory study

**DOI:** 10.1002/cam4.70010

**Published:** 2024-07-13

**Authors:** Timothy C. Guetterman, Rae Sakakibara, Srikar Baireddy, Wayne A. Babchuk

**Affiliations:** ^1^ University of Michigan Ann Arbor Michigan USA; ^2^ University of Nebraska‐Lincoln Lincoln Nebraska USA

**Keywords:** clinical cancer research, medical oncology, psychosocial studies, translational research

## Abstract

**Purpose:**

High‐quality communication is essential to patient‐centered care. Existing communication models and research tends to focus on what is said verbally with little attention to nonverbal aspects of communication. In sensitive and emotionally intensive healthcare encounters, such as in cancer care, provider and patient nonverbal behavior may be particularly important for communicating with empathy. Therefore, the aim of this study was to develop a conceptual model of communication that accounts for nonverbal behavior.

**Methods:**

We followed a systematic grounded theory design that involved semi‐structured interviews with 23 providers, including nurse practitioners, physicians, surgeons, and physician's assistants. Using constant comparative analysis, we analyzed transcripts and developed a grounded theory model of communication accounting for nonverbal behavior.

**Results:**

The major themes included building rapport, gauging how patients will take bad news, ensuring patients' understanding of their conditions, staying honest but hopeful, centering but guiding patient through cancer care, conveying empathy while managing heightened emotions, and ensuring patient understanding. Throughout the process, providers synthesize both verbal and nonverbal information and apply what they learn to future encounters.

**Conclusions:**

The results extend existing models of patient‐centered communication and invite communication intervention and research that incorporates nonverbal behavior. The model contributes an understanding of the full process of communication in clinical encounters.

## Graphical abstract

This qualitative study involved interviews with cancer care providers to develop a grounded theory model of communication that incorporates nonverbal and verbal behavior. The model reflects the importance of attending to nonverbals during encounters and has implications for future communication training and interventions.

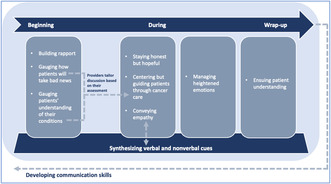

## 1 INTRODUCTION

High‐quality communication among patients and providers is a critical component of the healthcare encounter and is associated with fewer medical errors, fewer preventable sentinel events, decreased morbidity, reduced harm to patients, and increased patient activation and satisfaction.^1^, ^2^ Strong communication improves patient satisfaction and outcomes and is closely associated with patient‐centered care.^3^, ^4^, ^5^, ^6^ Research on healthcare communication has been heavily focused on verbal aspects, yet the body of research on nonverbal communication has provided evidence of its importance too. Nonverbal communication includes facial expressions, body positioning, and gestures—communication except for the actual language.^7^ Nonverbal behaviors contribute to perceptions of overall global affect, which is associated with patient satisfaction.^8^ The use of eye contact, gestures, and proximity to the patient can communicate the provider's interest in listening to the patient actively,^9^ which may be particularly true for historically minoritized patients, such as young Black women.^10^ Facing the patient, even when using a computer for notes, can improve patient‐provider communication and patient satisfaction in encounters.^11^, ^12^

Identifying and attending to the patients' nonverbal cues is also linked to improved patient satisfaction. Trials of training interventions to improve empathic communication by attending to nonverbal cues have demonstrated positive outcomes.^13^ However, less is known about how providers incorporate nonverbal behavior into clinical encounters in typical practice settings. Understanding providers experiences and perspectives with communication in potentially emotionally intensive encounters, such as communicating results or bad news to cancer patients, is needed to inform patient‐centered interventions.

**FIGURE 1:**
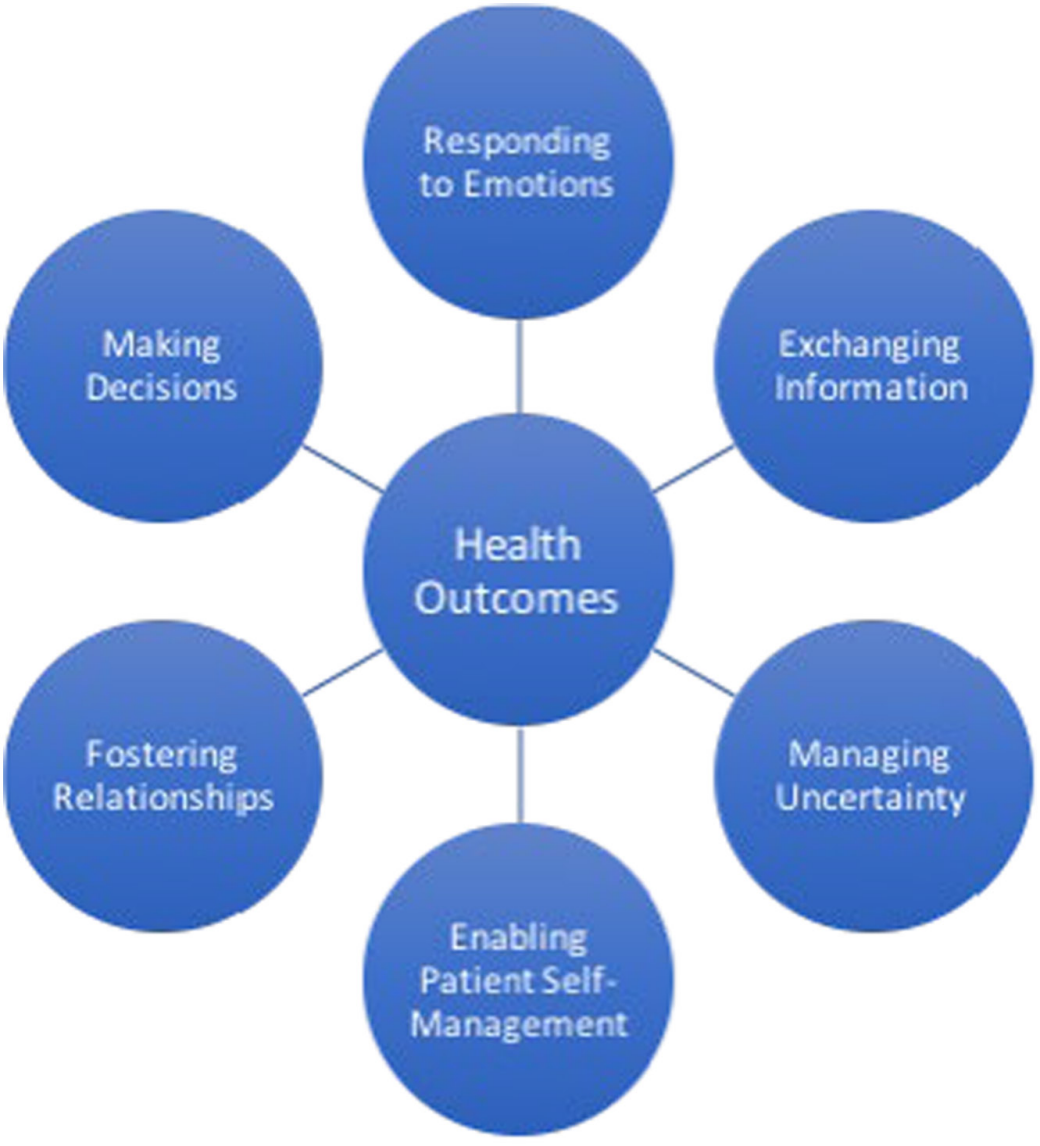
Conceptual model of core functions of patient‐centered communication. Adapted from Epstein & Street.^14^

The National Cancer Institute (NCI) framework of patient‐centered communication in cancer care^14^ provided a conceptual model for this study. The framework established the central role of communication in patient‐centered care (Figure 1). The framework conceives that health outcomes improve with attention to six core communication functions: (1) responding to emotions, (2) exchanging information, (3) making decisions, (4) fostering healing relationships, (5) enabling patient self‐management, and (6) managing uncertainty.^14^ Although not explicitly part of the framework, nonverbal behavior can be incorporated into these core functions for a more complete model of patient‐provider communication. However, providers' firsthand perspectives of intentional verbal in addition to nonverbal communication are needed to further refine the patient‐centered communication framework. Thus, the aim of this study was to develop a conceptual model of communication that accounts for nonverbal behavior, extending the patient‐centered communication in cancer care framework.

## 2 METHODS

We conducted a grounded theory study to develop a revised model communication in cancer care that accounts for both verbal and nonverbal behavior. Grounded theory was well‐suited to the intent of developing a model that explains the process of communication. Using Corbin and Strauss' systematic grounded theory design,^15^, ^16^ we sampled providers of cancer care, conducted semi‐structured interviews, and analyzed the data using grounded theory methods with the goal of developing a model of communication in the encounter.

### 2.1 Participants and sampling

Specifically, employing theoretical, purposeful sampling^15^ we recruited providers (i.e., physicians, nurse practitioners, physician assistants) who were actively seeing oncology patients using maximum variation sampling that seeks a breadth of participant characteristics. We recruited 23 providers from the healthcare organizations in the state of Michigan, seeking variation in terms of cancer specialty, years since training, and gender. We sent recruitment flyers via email asking providers to contact us if they were willing to participate. In addition, for recruitment purposes, we presented briefly at a grand rounds presentation within internal medicine to explain the study and ask for participants to contact us if they were willing to participate in an interview. The ultimate sample size was determined by theoretical saturation—the point at which the theory is developed and new information is not emerging nor adding complexity to the model.^17^, ^18^ We identified potential saturation at 20 interviews but continued with three additional individuals to challenge the point of saturation and concluded additional interviews were not adding complexity to findings.

### 2.2 Semi‐structured Interviews

We interviewed providers and focused on understanding intentional nonverbal communication (e.g., mirroring, silence, distance, smiling, nodding, leaning) and verbal communication. The framework of patient‐centered communication in cancer care^14^ informed our interview questions as we sought to probe providers regarding experiences in each of the core functions. Two of the authors (RS, TG) conducted semi‐structured interviews following a semi‐structured interview guide. Questions focused on approaches to communication and patient interaction, patient‐centeredness, nonverbal behavior, reciprocal communication, and training recommendations (Table 1). All interviews were professionally transcribed and de‐identified prior to analysis.

**TABLE 1:** Interview questions.

Tell me a bit about your clinical practice.
2 How do you approach communication with a patient?
3 What is your interaction like with your patient?
4 In what ways do you check the patient's understanding?
5 What would you consider as the most important aspects of patient‐centered communication?
6 (When you go into the patient room or meet virtually), how do you think about your nonverbal cues when interacting with patients? Do you think about it at all?
7 What have you noticed about patients' responses to your nonverbal behaviors?
8 How does the way in which you communicate affect your patients?
9 If someone asked you about how to train providers on one‐on‐one communication, what recommendations would you have?
10 What training recommendations would you have for providers regarding the nonverbal aspects of one‐on‐one communication?

### 2.3 Data analysis

We employed grounded theory analysis utilizing constant comparison,^15^, ^19^ beginning with open coding followed by axial and selective coding as outlined by Strauss and Corbin^20^ for the purpose of developing a theory of the process of patient‐provider nonverbal communication. All transcripts were de‐identified prior to analysis and imported into MAXQDA software for data management and analysis. Although we were aware of the framework for patient‐centered communication in cancer care,^14^ it did not explicitly inform our identification of codes. All codes identified were derived from the data. Three of the authors (RS, SB, and TG) conducting open coding of transcripts in MAXQDA and met to ensure agreement and consistency through a consensus process. We then employed axial coding to organize categories into the chronology of a visit. Next, in selective coding, we first identified a core category, synthesizing verbal and nonverbal cues, which represented how providers perceive and react to both verbal and nonverbal cues. Using MAXQDA, we then examined patterns among codes to identify themes and organize themes into a model. Through several iterations, we developed the final grounded theory three‐stage process model of communication presented below. We engaged in several procedures to check the validity of results. We conducted extensive searches for disconfirming evidence and in the results report instances of multiple, differing perspectives. In addition, we employed investigator triangulation by involving investigators with expertise in qualitative research, cancer communication, medicine, and health services research.

### 2.4 Ethical considerations

The University of Michigan Institutional Board (HUM00134766) granted ethical approval for the study prior to any data collection. Participants received invitations via email with the option of voluntary participation in the study. Because the initial study launch was during the initial period of the COVID‐19 pandemic, we delayed data collection by 1 year understanding that the healthcare providers we intended to invite had other personal and professional priorities that required their time and attention. All participants provided verbal consent and received an informed consent document with study information. Participants received an electronic gift card for their time participating in interviews. All interview transcripts were de‐identified to the extent possible, including removing names and mentions of specific organizations prior to importing into MAXQDA for analysis.

## 3 RESULTS

Participants included 23 providers, with approximately half physicians (*n* = 12) in addition to nurse practitioners (*n* = 6), and physician assistants (*n* = 5) as presented in Table 2. Reflecting the interactive nature of the communication process, the major themes were building rapport, gauging how patients will take bad news, ensuring patients' understanding of their conditions, staying honest but hopeful, centering but guiding patient through cancer care, conveying empathy while managing heightened emotions, and ensuring patient understanding.

**TABLE 2:** Summary of oncology provider characteristics.

Type of Provider	Frequency
Physician	12
Nurse Practitioner	6
Physician Assistant	5
Cancer specialization	
Breast cancer	2
Emergency oncology clinic	1
Genitourinary oncology	1
Gynecologic oncology	1
Hematology	5
Melanoma	1
Multiple Myeloma	1
Neurologic oncology	2
Neuroradiology	1
Medical oncology	1
Radiation oncology	1
Surgical oncology	3
Thoracic medical oncology	3
Typical Practice Setting	
Both	5
Outpatient	15
Inpatient	3
Years in Practice (mean)	17

### 3.1 Beginning: Building rapport, gauging how patients will take bad news, and assessing patient's understanding of their condition

The first category of themes focuses on the beginning of the encounter (Table 3). Building rapport begins from entering the exam room. A physician assistant described intentionality in entering the room consistently, “I try not to walk in the room already portraying doom and gloom if I know I have to give bad news. I try to always come in the room the same way for every patient, every time regardless of what we're going to talk about so that the patient does not try to read me before I'm ready to discuss” (P01, physician assistant, hematology). Providers described that toward the beginning of their visits they like to ask open‐ended questions about how their patients have been doing and how much they know about their condition. These questions assist to build rapport, gauge how the patient would take bad news, and assess how much more information they need to give. A provider explained, “I start the encounter with ‘can you tell me what your understanding of your situation is?’ And so then they summarize their clinical presentation. But also whatever they understand what the diagnosis and treatment options are. And a lot of times, that gives me a clue as to where they are, what they've accepted and what they haven't accepted. And sometimes they offer further explicit clues about where they are and how they're going to take it” (P02, surgeon, surgical oncology). Nonverbal cues are also important to gauge how patients will take news. A provider explained, “Well, for one, we can tell something by their eye movements, whether they are rolling up their eyes as I'm talking to them or they are trying to heave a sigh or they're anxiously trying to look away and not making eye contact” (P18, physician, neuroradiology).

**TABLE 3:** Themes related to nonverbal communication processes at the beginning of the encounter.

Theme	Illustrative Quote
Building rapport	“I try to use open body language. I try to be mindful to not cross my arms and my legs when I'm in the room.” (P15, physician's assistant) “And I'm sure that it shows on my face when I'm happy with labs or disappointed with labs or scans or things like that, walking in the room.” (P05, physician)
Gauging how patients Will take bad news	“And you get a feel for people pretty quickly when you're in that room, about what their level of anxiety is, what they can deal with, how far to push it, as far as being realistic with them. How much can wait till subsequent visits. You just get a feel for them, verbally and non‐verbally as well. And you tailor it to what they're leading.” (P12, physician) “Number one, it was how well I know the patient and they know me, like how long they've been under my care. I think that has two roles. One is, the more comfortable they are, they may be more comfortable allowing themselves to sort of emote, break down. At the same time, they may emote less because they know I'm here for them and that they have someone who was going to be here for them because I've been there for them before.” (P19, physician)
Assessing patient's understanding of their condition	“I guess, the thing that I found to be most useful is, especially with my new patients, I always ask them for what they understand so far about what was been going on in terms of what they've been told about the diagnosis, what they hear, our proposed treatments and the stage of the cancer. This is always, I think, helpful for me to understand where my patients are at and that helps me kind of frame my conversation.” (P09, physician) “I will pull up scans and use pictures to illustrate the tumor, what the tumor is close to, if the tumor is touching. And a lot of times, I think the picture communicates a lot of things that we're not able to communicate as well verbally. And so sometimes seeing the picture of the tumor and the relationship to the critical structures, I can often see an aha moment with the patient of understanding what they're facing.” (P02, surgical oncologist)

During the beginning of the encounter, providers also keenly focus on assessing patient's understanding of their diagnosis and condition. This time provides an essential check that will be repeated throughout the conversation while addressing patients' questions and discussing treatment plans. For example, a provider explained how they assess whether a patient is understanding: “Periodically, I will stop instead of just viewing a large amount of information all at one time. Maybe I'll explain their laboratory findings and then I'll ask them, ‘Do you understand this or do you have any questions?’ Then we'll go maybe onto the CT scan findings and we'll go through that and I'll make sure that they understand and give them the opportunity to ask questions” (P15, physician's assistant, gynecological oncology). Providers also assess for understanding through nonverbal behavior. A physician explained looking for nonverbal cues: “Or they're furrowing their brow, or they're… You know. Or they're a little listless at that time. So you know…There's something going on” (P07, physician, hematology). Then, based on their patients' stories, it seems providers like to tailor how much information to provide, taking into consideration both how much the patient can emotionally handle and how much they need to know based on their level of understanding. They are centering the patient while also guiding the conversation about cancer care, including their treatment plan options and how to move forward.

### 3.2 During: Staying honest but hopeful, centering and guiding patients through cancer care, conveying empathy, managing heightened emotions

Next, the themes center around the processes that occur during the encounter (Table 4). The providers discussed being intentional about communication during the visit. Most described their goal to be realistic and honest while instilling hope. A surgical oncologist explained, “I think I've had feedback that the patients appreciate that I am direct. I would say I'm not a false hope giver. I try to really accurately communicate whatever the situation is. And sometimes again, that's reassuring patients that they've got an excellent prognosis. But many times, it's being realistic about the risk of recurrence down the road and things like that” (P02, Surgeon, Surgical Oncology).

**TABLE 4:** Themes related to processes during the encounter.

Theme	Illustrative quote
Staying honest but hopeful	“I also feel strongly that cancer providers, like transplant providers too actually, a major part of our job is to provide hope for patients, even if that does not mean cure, so to give them clarity of thoughts about their diagnosis and a clear understanding of what is possible and what is not possible, and then a promise that you will walk that path with them as they make the decisions that they believe are right for them.” (P22, surgeon, surgical oncology) “Especially in my world of prostate cancer, there was many treatment options. We try to present them in a very unbiased manner. Basically, letting them know that we're not God, we have no crystal ball, what we are good at is giving probabilities, what we are not good at is giving certainties…Trying to make sure they understand the risks and the benefits that we're talking about and for them to sort of interject throughout with questions.” (P19, radiation oncology)
Centering and guiding patients through cancer care	“I definitely make sure to always keep eye contact with them. I know that some docs, because you know how busy we are, like to type on the computer during a patient encounter.” I never do that. I rarely try to use the computer. If I am using the computer, I say, “I'm just looking up your labs or just looking up your scan results and giving you the details.” (P09, physician, thoracic oncology) “…assuring them that, ‘Look, we're not going to make or break treatment whether we start this week or next week,’ which is usually the case in the vast majority of patients.” So assuring them, because they're scared and they're like, ‘Well, I'm not sure I know what I want to do, but I don't want to delay treatment either.’ And so trying to give them some assurance and say, ‘Look, think about this. Talk with your family. Then we'll plan to get the show on the road next week.’ And again, usually that gives them time to, okay, go home and think about it and talk to their children and their spouse, and then they come back and say, “Yeah, we discussed this. This is what we want to do.” (P10, physician, thoracic oncology)
Conveying empathy, managing heightened emotions	“For the majority, if there was a frustration about something or a heightened level of anxiety about something, if I am able to acknowledge their perspective and their emotion in it, but also give direct feedback within it and also be calm and respectful through the process and give them a little bit of space to express whatever it is so that they feel heard. That was another key piece is that sometimes people are just very frustrated because they don't feel that their experience is being understood or heard. And so to give them space to express that, sometimes that is all that is needed even if the situation doesn't even change.” (P04, nurse practitioner, hematology) “So when I notice that the patient is in distress, I will oftentimes lean towards them to kind of make them feel like I'm really hearing what they're saying.” (P01, physician's assistant, hematology)

In centering the patient, discussions involve goals of care and focus on shared decision‐making. Through the conversation, many providers described shared decision‐making models. A medical oncologist said, “But because the stakes are so high, I talk with the trainees. I say, ‘We can't be paternalistic. It was not like an antibiotic because there was real risk involved and you can't push something on them, because then you're the one that was held responsible.’ Then when things go bad, they say, ‘Well, I didn't want any of this anyway but Dr. [name redacted] forced me to do this and now I'm worse off.’ So that shared decision making is critical” (P10, Physician, thoracic medical oncology).

Providers also described ways in which they convey empathy to patients through what they say in addition to nonverbal cues. Providers described their awareness that patients may have already had extensive evaluation through primary care or other cancer providers, and some patients simply need time and space to express their feelings. Some providers described how they incorporate nonverbal behavior to convey empathy. A surgical oncologist explained their deliberate body positioning, “I'd say the biggest nonverbal clue I try to give is that I'm not in a hurry. I sit down, I don't stand by the door, and just really try to communicate that I'm there to answer any and all questions that they have” (P02, surgeon, surgical oncology). Many providers discussed their goal to remain empathetic throughout the encounter and to display sensitivity if patients become upset. A nurse practitioner said, “I always say, I'm sorry I have to tell you this” (P11, nurse practitioner, neurological oncology).

Patients' emotions are often heightened during encounters concerning delivering bad news or in anticipation of news. Providers described patients as being understandably “anxious,” “upset,” or “wired.” They may pause the conversation or offer a break. A surgeon has, “asked patients if they want five or 10 min with their family members, which I guess somewhat to my surprise, patients don't usually take you up on” (P22, surgeon, liver surgery). Nonverbally, providers observed patients' behaviors for cues of heightened emotions. A nurse practitioner in surgical oncology with over 30 years of experience looks for nonverbal cues and responds accordingly: “When I see them start to get anxious, and their body's getting more rigid, or they start getting tears in their eyes, I'm definitely backing off, and just spending more time, and pausing more in between my discussion to give them time” (P06, nurse practitioner, surgical oncology‐melanoma, sarcoma). Providers also described attending to body positioning by facing the patient and maintaining eye contact as helpful in conveying empathy through heightened emotions. Several providers described watching for nonverbal cues include furrowing brows, narrowing eyes, scrunching their face, or even exchanging looks with others in the room. A physician's assistant specialized in hematology explained: “When I notice that the patient is in distress, I will oftentimes lean towards them to kind of make them feel like I'm really hearing what they're saying” (P01, physician assistant, hematology). Sitting at the same level of the patient in a chair was a strategy to manage emotions. When a patient becomes upset, providers may follow‐up with referrals to social work for additional support.

### 3.3 Wrap‐up: Ensuring patient understanding

The next theme of the process is ensuring patient understanding during the visit wrap‐up (Table 5). Checking in on patient understanding is again helpful as the encounter is concluding. Providers tended to describe checking for understanding throughout the visit but stressed its importance in closing. Several described strategies, such as a teachback by asking the patient to summarize the plan based on their understating or visual aids through drawings or diagrams. A nurse practitioner in hematology gives time for questions about the plan: “just asking them how they're doing and at the end of the visit, regardless of what you've discussed again asking like, so this is the plan. Does that sound good to you? Do you have any additional questions or concerns?” (P04, nurse practitioner, hematology). Some providers gauge facial expressions, such as whether the patient appears confused, and follow up with questions, information, or support accordingly. Reflecting on the conversation can be important in wrapping up. A surgeon described self‐awareness of “what I haven't explained well, or what they don't understand,” and if needed, asking questions about their personal lives and goals to “bring their focus back because I think they can glaze over when you're talking about the nuances of a particular diagnosis” (P22, surgeon, liver surgery).

**TABLE 5:** Theme related to the Encounter wrap‐up.

Theme	Illustrative quote
Ensuring patient understanding	“I can show them the written paper scan. I get right next to them, and we read it together.” And I say, “I will translate this stuff for you in normal human terms, and then you ask me questions.” (P06, nurse practitioner, surgical oncology) “For some things, especially if I talk to you about a treatment or procedure or stuff like that, I will ask them to repeat back what their understanding is of what we just talked about.” (P16, physician, hematology)

### 3.4 Grounded theory‐derived model

**FIGURE 2:**
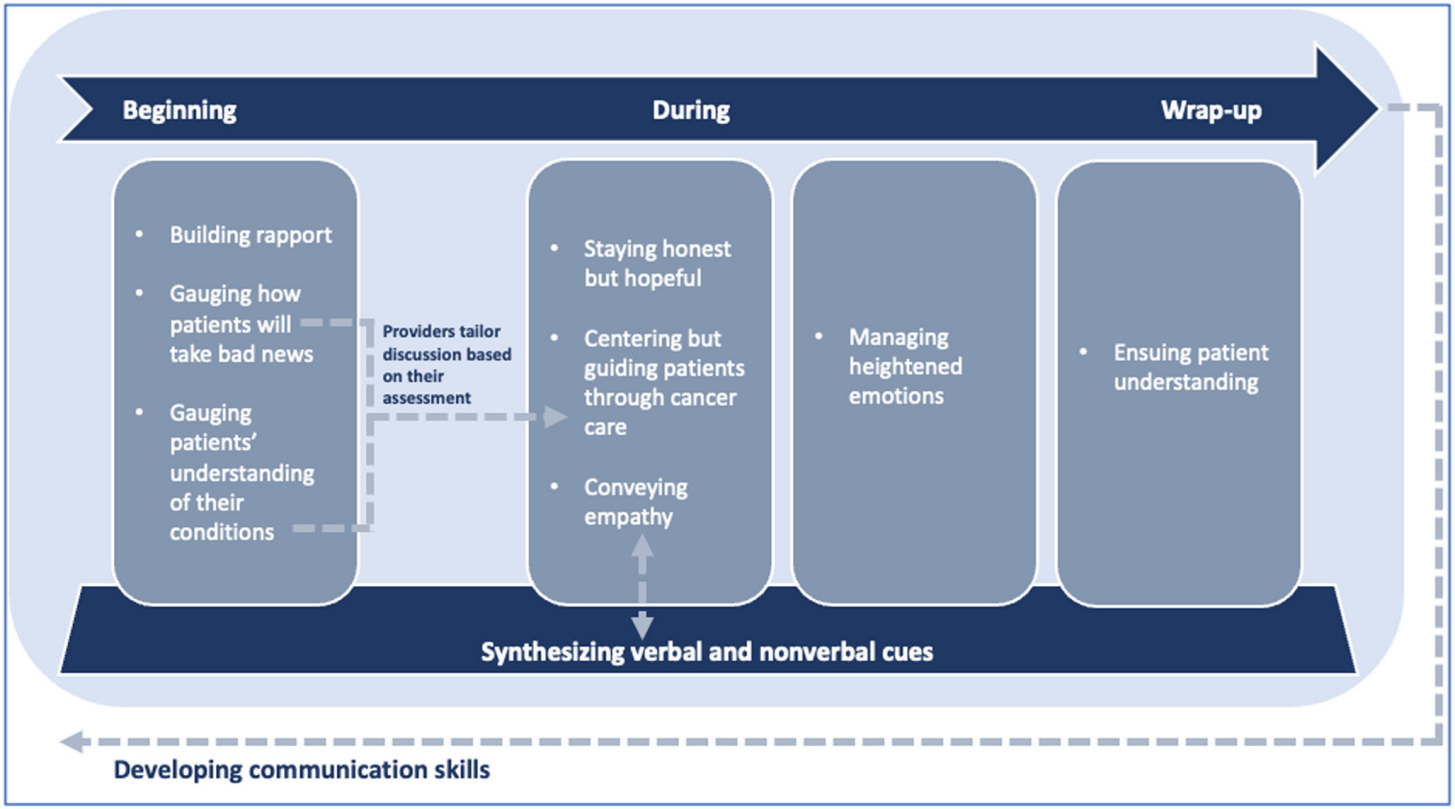
Grounded theory model of patient communication in cancer care incorporating verbal and nonverbal aspects.

Because communication through an encounter is a process, we organized the themes based on what occurs at the beginning of a visit, during the visit, and at wrap‐up to develop a model of patient communication in cancer care incorporating verbal and nonverbal aspects (Figure 2). Throughout the process, providers synthesized verbal and nonverbal question and behavior when conveying empathy. Each process cycle is iterative, repeating as providers develop their own communication skills. In developing the model, we separated the “ensuring patient understanding” code into “gaging patients' understanding of their condition” and “gaging patient understanding.” Because gaging how patients will take bad news and patients' understanding are related to each other and to centering but guiding through care, they are connected with a dotted arrow. In addition, based on the grounded theory analysis, we added two additional concepts: synthesizing verbal and nonverbal cues and developing communication skills. Certainly, external factors such as the health system can also shape communication, but our focus was on the patients and providers as individuals.

#### 3.4.1 Synthesizing verbal and nonverbal cues

At the base of the model is the “synthesizing verbal and nonverbal cues” theme. The results indicate how providers are observing and using patients' nonverbal cues including their physical appearance and verbal question to help place their patients within their specific life context, outside of the office setting. Synthesizing all this information helps providers understand their patients' treatment goals and how to approach cancer care communication. Synthesizing verbal and nonverbal cues is the foundation on which good communication is built, so it rests at the bottom in the model. This theme is connected with a bidirectional dotted arrow to “conveying empathy” specifically because providers noted how they try to convey empathy by understanding how patients' cancer diagnoses affect their lives and tailor their care to their individual needs. A nurse practitioner explained, “It's all listening. The nonverbal help. If they don't look you in the eye, if they don't let you get your questions in because they want to talk and tell you what they want, or what they think” (P11, nurse practitioner, neurooncology). On the other hand, several providers also described their own nonverbal behavior, such as how they may present when they are nervous. A physician assistant described this awareness at an early career point, “for example, I have a really obnoxious thing that when I'm nervous, I giggle…I didn't even know I had that weird reaction until I got into this field” (P01, physician assistant, hematology).

However, despite our aim to focus on nonverbal communication and elicit responses through specific questions, probes, and follow‐up questions, a major finding was that providers were not often aware of their patients or their own nonverbal behavior. Asked about patients' responses to their own body language, an experienced physician replied authentically, “I don't know that I can answer that question” (P03, physician, medical oncology).

#### 3.4.2 Developing communication skills

The final aspect of the model is “developing communication skills,” because providers noted that they mostly learn effective skills through experience. In that way, this theme is related to all themes and represents a cyclical process of continuous improvement.

## 4 DISCUSSION

The results of the analysis provide a grounded theory model of communication in cancer care, which often involves breaking bad news and attending to understandably strong emotions. The consideration of both verbal and nonverbal communication is relatively novel and extends existing models of communication. Clinical implications include the importance of attending to both nonverbal and verbal behavior during healthcare visits. Our results indicated that providers were not often cognizant of their nonverbal behavior nor how they interpret patient's nonverbal behavior although the questions prompted them to consider it in depth. The results provide a framework to think about and attend to nonverbal communication throughout an encounter, which is particularly important given the role of nonverbal behavior in building rapport, improving patient satisfaction, and enhancing patient engagement.^8^, ^10^, ^11^, ^12^

We returned to the framework for patient‐centered communication cancer care in interpretation of our results and interrogated how our model of patient communication in cancer care builds on the framework. Many of the key aspects in the framework were reflected in the grounded theory model we developed. The main tenets of the framework are: (1) responding to emotions, (2) exchanging information, (3) making decisions, (4) fostering healing relationships, (5) enabling patient self‐management, and (6) managing uncertainty. Responding to emotions maps on to conveying empathy and managing heightened emotions. Conveying empathy involves demonstrating a shared understanding with the patient and supporting the patient through difficulties.^21^ Exchanging information is consistent with our finding of centering but guiding patients and ensuring patient understanding and further supports patient and caregiver desire for information about options.^22^ Finally, fostering healing relationships is supported by our finding of building rapport, conveying empathy, and synthesizing verbal and nonverbal cues. A key difference is that our model, derived from views of cancer care providers, is organized into a process model from beginning to visit wrap‐up along with an integrative process in which providers learn from each visit. Future research should also explore cultural differences in verbal and nonverbal communication. Nonverbal norms and behaviors may vary given cultural differences and be particularly important when providers and patients are from different racial, ethnic, or cultural backgrounds.^23^, ^24^, ^25^ Even among the generally agreed emotional expressions (e.g., happiness, sadness, surprise, disgust, fear, and anger), how those nonverbal expression are perceived in interactions differs across cultures.^26^

The present research extends the existing models by organizing important aspects of verbal and nonverbal communication into the process of an encounter. While the framework for patient‐centered communication cancer care is broadly applicable to patient‐centeredness in managing cancer care, the grounded theory model developed is specific to what occurs during visits. Yet, between visits, providers described ongoing learning by transferring lessons from each encounter to future encounters with other patients. The period between visits may be an optimal period for interventions, such as guided debriefing with a peer or mentor, that considers verbal and nonverbal aspects.^27^, ^28^ Because providers interviewed were often less aware of their nonverbal behavior, additional interventions might use methods such as reviewing snippets of video encounters as an elicitation technique for debriefing.

Other existing models focused on structured conversation advice for clinical providers, including goals of care and breaking bad news. The serious illness conversation guide enables conversations about goals and values and was developed through a community‐engaged process.^29^ Evidence indicates the guide is valuable in improving conversations and strengthening relationships.^30^ Similar to our model of patient communication in cancer care incorporating verbal and nonverbal aspects, the serious illness conversation guide follows the encounter process—set up, assess, share, explore, and close. Similarly, REMAP (Reframe, Expect emotion, Map out patient goals, Align with goals, Propose a plan) also focuses on goals of care and does briefly address nonverbal expressions of emotion.^31^ The SPIKES protocol provides specific verbal steps through a conversation but is focused on breaking bad news.^32^ SPIKES includes the setup, patient perception, an invitation from patients for information, imparting information, addressing emotions, and leaving with a summary and strategy.^32^ However, our model of patient communication differs in that it is broader and focused on the entire encounter. Although our results included experiences of delivering bad news, providers also shared experiences with delivering good news and more neutral encounters.

A limitation of this research is that it does not currently account for patient views. Future research should examine patient perspectives because communication is a reciprocal and interactive process. Many participants seemed keenly aware of what they said and what patients said verbally yet had given less thought to nonverbal aspects. Additional research might rely on elicitation techniques with providers, such as reviewing snippets of recorded encounters or debriefing immediately after an encounter. These methods may better prime providers to think about nonverbal behavior. We were, however, able to incorporate nonverbal aspects that advances existing models. In addition, we did not collect data about providers ethnicity, gender, or age, which is important in considering concordance and patient‐provider communication. A final limitation is that this model provides an initial grounded theory model. The model needs to be verified and tested through additional quantitative or mixed methods research.

## 5 CONCLUSION

The study was prompted by the need to incorporate nonverbal behavior into communication simulation and interventions, which has been largely absent. The results may inform interventions to improve providers' dissemination of health information and related health outcomes for patients through an enhanced conceptual model of patient‐centered communication that not only describes core communication functions but also incorporates nonverbal expressions into a model of the interrelationships between those components.

## AUTHOR CONTRIBUTIONS

**Timothy C. Guetterman:** Conceptualization (lead); data curation (lead); formal analysis (lead); funding acquisition (lead); investigation (lead); methodology (lead); project administration (lead); resources (lead); software (lead); supervision (lead); validation (lead); visualization (lead); writing – original draft (lead); writing – review and editing (lead). **Rae Sakakibara:** Data curation (equal); formal analysis (equal); project administration (lead); writing – original draft (supporting); writing – review and editing (supporting). **Srikar Baireddy:** Data curation (supporting); formal analysis (supporting); software (supporting); writing – original draft (supporting); writing – review and editing (supporting). **Wayne A. Babchuk:** Conceptualization (supporting); methodology (supporting); writing – original draft (supporting); writing – review and editing (supporting).

## FUNDING INFORMATION

Funding was supported from the National Institutes of Health/National Library of Medicine (1‐K01‐LM‐012739).

## CONFLICT OF INTEREST STATEMENT

The author(s) declared no potential conflicts of interest with respect to the research, authorship, and/or publication of this article.

## INFORMED CONSENT

All participants of the study received an informed consent document and had an opportunity to ask questions directly to study investigators. Signatures were not collected.

## Data Availability

De‐identified data is available upon reasonable request.
